# Molecular Surveillance and *Ex Vivo* Drug Susceptibilities of *Plasmodium vivax* Isolates From the China–Myanmar Border

**DOI:** 10.3389/fcimb.2021.738075

**Published:** 2021-11-01

**Authors:** Weilin Zeng, Hui Zhao, Wei Zhao, Qi Yang, Xinxin Li, Xiaosong Li, Mengxi Duan, Xun Wang, Cuiying Li, Zheng Xiang, Xi Chen, Liwang Cui, Zhaoqing Yang

**Affiliations:** ^1^ Department of Pathogen Biology and Immunology, Kunming Medical University, Kunming, China; ^2^ Department of Internal Medicine, Morsani College of Medicine, University of South Florida, Tampa, FL, United States

**Keywords:** *Plasmodium vivax*, drug sensitivity, multidrug resistance-1 gene, multidrug resistance protein 1 gene, China–Myanmar border

## Abstract

Drug resistance in *Plasmodium vivax* may pose a challenge to malaria elimination. Previous studies have found that *P. vivax* has a decreased sensitivity to antimalarial drugs in some areas of the Greater Mekong Sub-region. This study aims to investigate the *ex vivo* drug susceptibilities of *P*. *vivax* isolates from the China–Myanmar border and genetic variations of resistance-related genes. A total of 46 P. *vivax* clinical isolates were assessed for *ex vivo* susceptibility to seven antimalarial drugs using the schizont maturation assay. The medians of IC_50_ (half-maximum inhibitory concentrations) for chloroquine, artesunate, and dihydroartemisinin from 46 parasite isolates were 96.48, 1.95, and 1.63 nM, respectively, while the medians of IC_50_ values for piperaquine, pyronaridine, mefloquine, and quinine from 39 parasite isolates were 19.60, 15.53, 16.38, and 26.04 nM, respectively. Sequence polymorphisms in *pvmdr1* (*P*. *vivax* multidrug resistance-1), *pvmrp1* (*P*. *vivax* multidrug resistance protein 1), *pvdhfr* (*P*. *vivax* dihydrofolate reductase), and *pvdhps* (*P*. *vivax* dihydropteroate synthase) were determined by PCR and sequencing. *Pvmdr1* had 13 non-synonymous substitutions, of which, T908S and T958M were fixed, G698S (97.8%) and F1076L (93.5%) were highly prevalent, and other substitutions had relatively low prevalences. *Pvmrp1* had three non-synonymous substitutions, with Y1393D being fixed, G1419A approaching fixation (97.8%), and V1478I being rare (2.2%). Several *pvdhfr* and *pvdhps* mutations were relatively frequent in the studied parasite population. The *pvmdr1* G698S substitution was associated with a reduced sensitivity to chloroquine, artesunate, and dihydroartemisinin. This study suggests the possible emergence of *P*. *vivax* isolates resistant to certain antimalarial drugs at the China–Myanmar border, which demands continuous surveillance for drug resistance.

## Introduction

*Plasmodium vivax* is the most widely distributed malaria parasite, which can also cause life-threatening diseases. *P. vivax* was responsible for 6.4 million cases globally in 2019, with nearly 51.7% of the cases in Southeast Asia (WHO, 2020). Countries within the Greater Mekong Sub-region (GMS) are pursuing malaria elimination by 2030 (WHO, 2015). As the GMS is progressing toward eliminating *P. falciparum* by 2025, the proportion of malaria cases caused by *P. vivax* infections has increased steadily. Myanmar carries the heaviest malaria burden, with malaria transmission concentrated along its international borders (Geng et al., 2019). In most vivax-endemic areas, the combination of chloroquine (CQ) and primaquine remains the first-line treatment for uncomplicated *P. vivax*, but CQ resistance has been an increasing concern. Although artemisinin-based combination therapies (ACTs) as a unified treatment of *P. falciparum* and *P. vivax* infection may have significant advantages in areas where both species coexist (Douglas et al., 2010), ACT treatment of vivax malaria is commonly deployed in areas such as Indonesia, where *P. vivax* CQ resistance was evident (Baird, 2011).

CQ was first produced in 1934 and soon proved to be one of the most successful and important antimalarial drugs (Wellems and Plowe, 2001). However, the extensive use of CQ in the following decades eventually led to drug resistance. *P. falciparum* developed resistance in various areas since the 1950s, but drug-resistant *P. vivax* was not reported until the 1980s in Indonesia and Papua New Guinea (Rieckmann et al., 1989; Baird et al., 1991). To date, CQ-resistant (CQR) *P. vivax* has been detected in more than ten countries (Price et al., 2014). As early as 1993, CQR *P. vivax* cases were reported in Myanmar (Myat Phone et al., 1993; Marlar et al., 1995; Guthmann et al., 2008). In recent years, clinical failures after CQ treatment, indicating RI or even RII type resistance as defined by the WHO criteria (WHO, 1967), have been reported in many regions of Myanmar (Yuan et al., 2015; Htun et al., 2017; Xu et al., 2020). At the Thai–Myanmar border, 8% of *P. vivax* patients developed recurrent parasitemia within 28 days after CQ treatment (Chu et al., 2018). In addition, although the recurrence rate of *P. vivax* infection within 28 days after CQ treatment in other parts of the GMS such as Vietnam (3.5%) and western Thailand (8%) was still low (Rijken et al., 2011; Amaratunga et al., 2014a), CQR *P*. *vivax* isolates were confirmed in these areas (Rijken et al., 2011; Thanh et al., 2015). Therefore, the CQR parasites have become a focus of attention in the GMS, especially since this parasite is targeted for elimination.

Sulfadoxine-pyrimethamine (SP) has a long history of combating malaria due to its safety, good tolerance, and long-lasting activity (Müller and Hyde, 2013). In China, as a component of the two combination prophylactic regimens, SP was widely used for malaria prophylaxis between the mid-1960s and early 1990s (Huang et al., 2014). Under this scenario, all malaria parasite species should be under a selection pressure by antifolate drugs. In addition, although SP has rarely been used to treat vivax malaria, the prevalence of mixed *P. falciparum/P. vivax* infections in the region (Li et al., 2014) suggests that *P. vivax* might also be under selection by antifolate drugs used to treat *P. falciparum* infections. A decade ago, *P. falciparum* resistance to artemisinin family drugs was detected first in Cambodia (Noedl et al., 2008; Amaratunga et al., 2014b) and then in other countries of the GMS (Holmgren et al., 2007; Dondorp et al., 2009; Ashley et al., 2014). *P. falciparum* has also developed resistance to ACT partner drugs such as mefloquine (MFQ) and piperaquine (PPQ) (Price et al., 2004; Nawaz et al., 2009; Cui et al., 2012). Cross-sectional surveys in the Thai–Myanmar border area, Cambodia and Vietnam detected 1% of infections as mixed *P. falciparum/P. vivax* infections (Imwong et al., 2015). This rate reached 14% among febrile patients at the China–Myanmar border. Furthermore, misdiagnosis of malaria cases is also common; a recent survey of acute malaria cases showed that as much as 20% of *P. vivax* cases were misdiagnosed in the field (Geng et al., 2019). These studies highlight the inevitable collateral selection of *P. vivax* by the commonly used antimalarial drugs used to treat *P. falciparum*.

The underlying mechanisms of antimalarial drug resistance in *P. vivax* are less well understood than those in *P. falciparum*. *P. vivax* CQR mechanism appears different from that for *P. falciparum*. Mutations in the *P. falciparum* chloroquine resistance transporter (*pfcrt*) are the main determinants of CQ resistance (Fidock et al., 2000), but studies failed to identify an association between CQ resistance and mutations in the *pfcrt* ortholog, *P. vivax* chloroquine resistance transporter-o (*pvcrt-o*) (Nomura et al., 2001). The *pfmdr1* (*P*. *falciparum multidrug resistance 1*) gene is often used to monitor the resistance of *P. falciparum* to 4-aminoquinolines and MFQ (Duraisingh et al., 2000; Djimdé et al., 2001; Price et al., 2004; Happi et al., 2006). Its ortholog in *P*. *vivax*, *pvmdr1*, may also play a role in CQ resistance. Some identified an association of the *pvmdr1* Y976F substitution with CQR *P*. *vivax in vitro* (Suwanarusk et al., 2007), but others did not find such an association (Sá et al., 2005; Barnadas et al., 2008; Gomes et al., 2016). The *P*. *falciparum multidrug resistance protein 1* (*pfmrp1*) gene, encoding an ABC transporter transmembrane protein, is a potential multidrug-resistance candidate in *P. falciparum* with mutations associated with a reduced susceptibility to CQ (Veiga et al., 2011; Gupta et al., 2014). Therefore, although several genes are suspected to be associated with CQ resistance in *P. vivax*, there are no definitive molecular markers for monitoring *P. vivax* CQ resistance. Mutations in *dihydrofolate reductase* (*pvdhfr*) and *dihydropteroate synthase* (*pvdhps*) have been associated with the altered clinical response to SP (de Pécoulas et al., 1998; Eldin de Pécoulas et al., 1998; Imwong et al., 2001; Tjitra et al., 2002; Imwong et al., 2003; Korsinczky et al., 2004). To track the *ex vivo* drug susceptibilities of *P. vivax* parasites in northeast Myanmar, we collected clinical samples and profiled their *ex vivo* susceptibilities to seven antimalarial drugs, which are the first-line drugs for treating vivax or falciparum malaria in the GMS. Furthermore, we surveyed the polymorphisms of candidate resistance markers *pvmdr1*, *pvmrp1*, *pvdhfr*, and *pvdhps* in these isolates.

## Material and Methods

### Ethical Approvals, Study Site, and Parasite Isolates

This study was approved by the institutional review boards of Kunming Medical University and Pennsylvania State University. Signed informed consent/assent forms were obtained from all patients and guardians in the case of minors. *P. vivax* clinical isolates were obtained from *P. vivax* patients aged seven years and older who attended the malaria clinics located at the Laiza town, Kachin State, Myanmar, in May and July of 2016. Patients with clinical symptoms of malaria (e.g., rigor, fever, headache, malaise, muscle pains) were diagnosed by a microscopic examination of Giemsa-stained blood smears. Parasites were staged based on morphological characteristics described earlier (Russell et al., 2003). Only patients with >0.5% parasitemia and >70% ring stage on presentation and without taking antimalarial medicine were recruited. Patients younger than 7 years, anemic patients (hemoglobin level <7 g/dl), pregnant and lactating women, and those who took antimalarial drugs in the preceding two weeks were excluded. Three (for patients aged 7–17 years) or five milliliters (for patients aged ≥18 years) of blood were drawn from each patient by venipuncture into a heparin-coated tube, transported to the nearby laboratory in a 37°C thermos, and used for *ex vivo* drug assays within four hours of collection.

### *Ex Vivo* Drug Assay

CQ, MFQ, quinine (QN), and pyronaridine phosphate (PND) were obtained from Sigma. PPQ was obtained from Chongqing Kangle Pharmaceutical Co., Ltd (Chongqing, China). Dihydroartemisinin (DHA) and artesunate (AS) were obtained from Kunming Pharmaceutical Corp. (Kunming, China). The stock solutions and serial dilutions were made according to the previous description (Li et al., 2020). Each drug concentration was tested in triplicate. The testing plates were dried inside a biosafety hood, sealed, and stored at 4°C for use within two weeks. The *ex vivo* drug assay, i.e., the schizont maturation assay, was performed as described earlier (Li et al., 2017; Li et al., 2020). Two milliliters of *P*. *vivax*-infected blood were centrifuged at 1,000 rpm for 10 min, and the cell pellet was resuspended with two volumes of RPMI 1640. Leukocytes were removed using an NWF filter (Zhi Xing Bio, Bengbu, China) (Tao et al., 2011). Then, 400 μL of the packed erythrocytes were resuspended in 19.6 mL of culture medium containing McCoy’s 5A (11.9 g/L, Sigma, St. Louis, MO, USA), HEPES (25 ml/L, Sigma), gentamicin (5 mg/L, Jinan Limin Pharmaceutical Co., Ltd, Jinan, China), 7.5% NaHCO3 (2.1 g/L, Sigma), and 25% AB+ serum from malaria-naive donors to make a 2% hematocrit. The red blood cell (RBC) suspension (100 μL/well) was dispensed into the wells of a Costar 96-well flat-bottom microtiter plate (Sigma), which was pre-dosed with antimalarial drugs. The plates were then incubated in a candle jar at 37°C for 26–36 h depending on the stage of the parasites. Twelve hours later, parasite development in the drug-free wells was monitored every 1–4 h. When more than 40% of the parasites in the control wells developed to the mature schizont stage, all wells were collected to prepare thick and thin films (Lu et al., 2012). Blood films were fixed with methanol, stained with 10% Giemsa (Sigma) solution for 30 min, and examined microscopically under oil immersion. Schizonts with at least three well-defined chromatin dots were counted. The number of schizonts per 200 asexual parasites at each drug concentration was determined and normalized with the control well.

### DNA Amplification and Sequencing

QiAamp DNA Mini Kit (Qiagen, Hilden, Germany) was used to extract parasite DNA from 200 μL of whole blood. Single-species *P*. *vivax* infections were confirmed by nested PCR targeting the 18S rRNA gene (Snounou et al., 1993). Then, *P*. *vivax merozoite surface protein- 3α* (*pvmsp-3α*) was genotyped using a PCR and restriction fragment length polymorphism (RFLP) protocol to confirm monoclonal *P. vivax* infections (Bruce et al., 1999). Multiple molecular markers, including *pvmdr1*, *pvmrp1*, *pvdhps*, and *pvdhfr*, which are related to drug resistance in *P. vivax*, were amplified and sequenced. The full-length *pvmdr1* (4,395 bp) was amplified by PCR using Phanta Max Super-Fidelity DNA polymerase P505d (Nanjing Vazyme Biotech, China) containing high-fidelity Pfu DNA polymerase with primers *pvmdr1*F (5′- CAGCAGACACCATTTAAGG-3′) and *pvmdr1*R (5′- CCGTTTGTTGATTAGTTGC-3′). Fragments of *pvmrp1* (497 bp), *pvdhps* (767 bp), and *pvdhfr* (632 bp), which covered potential drug resistance-associated mutations, were amplified by nested PCR using primers reported from previous studies (Mint Lekweiry et al., 2012; Chehuan et al., 2013) (**Supplementary Table S2**). All reactions were performed in 25 μL containing 12.5 μL of 2× Phanta Max Buffer, 10 mM of each dNTP mix, 0.5 μM of each primer, 0.5 U of Super-Fidelity DNA polymerase P505d, and 1 μL (5–10 ng) of genomic DNA. The PCR cycling parameters were as follows: initial denaturation at 95°C for 3 min, followed by 35 cycles at 95°C for 15 sec, 58°C for 15 sec, 72°C for 3 min, and then a final extension at 72°C for 5 min. The PCR products were resolved on a 1.2% agarose gel, and the sizes of the PCR products were determined using LD2000 or LD5000 DNA ladder (TaKaRa, Japan). Amplification products were sequenced on both strands by Sangon Biotech Co. Ltd. (Shanghai, China). Sequences were assembled using the SeqMan program of the Lasergene software (DNASTAR, Madison, WI, USA). Individual sequences were aligned to the Salvador I strain reference sequences retrieved from the GenBank (XM_001613678 for *pvmdr1*, XP_001612680 for *pvmrp1*, XP_001617209 for *pvdhps*, and XP_001615082 for *pvdhfr*) to determine the presence of single nucleotide polymorphisms in the respective genes. Nucleotide sequences were translated into amino acid sequences to examine mutant codons using BioEdit (version 7.2.5).

### Data Analysis

Statistical analyses were performed using GraphPad Prism6 (GraphPad Software, Inc, San Diego, CA, USA). Geometric means of the *ex vivo* IC_50_s (half-maximum inhibitory concentrations) were calculated by fitting the drug response data to a sigmoid curve. Medians and interquartile ranges (IQRs) were calculated because the data were not normally distributed. IC_50_s between groups were compared by using the Mann-Whitney U test and the Wilcoxon matched-pairs signed-rank test. Correlations between IC_50_s of drugs were determined using Spearman’s test in the R package.

## Results

### *Ex Vivo* Susceptibilities of Parasite Isolates to Antimalarial Drugs

**Figure 1:**
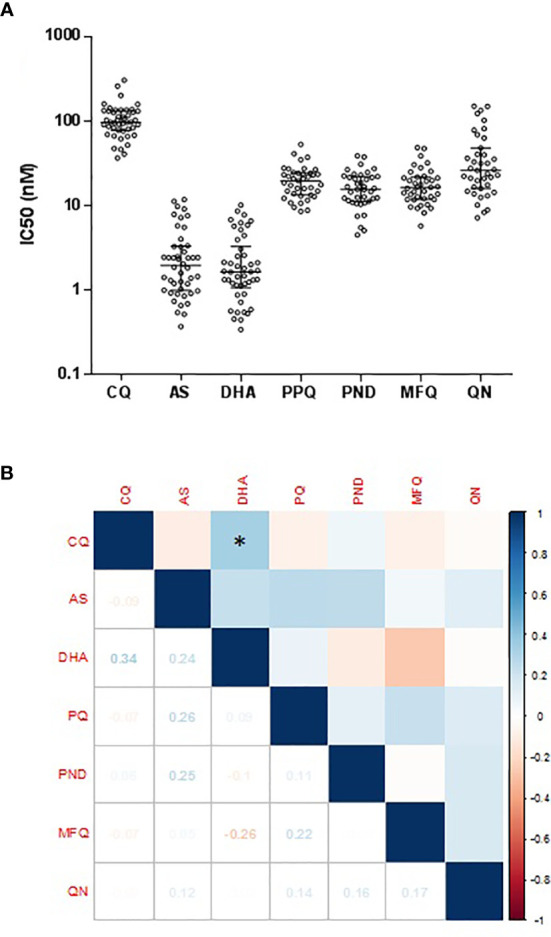
IC50 values of each antimalarial drug among *P. vivax* isolates *P. vivax* isolates for each antimalarial drug. **(A)** Dot plots of *ex vivo* susceptibilities of *P. vivax* isolates to seven antimalarial drugs. Comparison of *ex vivo* IC_50_ values (in nM) to seven antimalarial drugs. **(B)** Correlations between IC_50_s of *P. vivax* isolates to seven antimalarial drugs. Used Spearman’s test to analyze the correlation between IC_50_s Spearman’s correlation analysis between IC_50_ values of parasite isolates to seven antimalarial drugs. The values of correlation coefficient based on the right side of the color scale. The values of the correlation coefficient were based on the color scale shown on the right. *indicates a significant correlation.

Parasite samples were obtained from 97 P*. vivax* patients fitting the recruitment criteria and were used to determine susceptibilities to seven antimalarial drugs. Among them, 57 isolates successfully developed to mature schizonts. Genotyping by RFLP analysis of *pvmsp3α* determined that 46 were monoclonal *P. vivax* infections. All 46 isolates were successfully assayed for CQ, AS, and DHA. Since seven samples were not sufficient for assaying all drugs, 39 isolates were assayed for PPQ, PND, MFQ, and QN (**Table 1** and **Figure 1A**). For CQ, the median *ex vivo* IC_50_ was 96.48 nM (range: 36.5–303.4 nM), with 4.4% (2/46) isolates having IC_50_ values above 220 nM. For the artemisinin derivatives AS and DHA, the median IC_50_ was 1.95 (range: 0.4–11.8 nM) and 1.63 (range: 0.3–10.2 nM), respectively. For PND, the median IC_50_ was 15.5 nM (range: 4.5–38.8 nM). For MFQ, the median IC_50_ was 16.4 nM (range: 5.8–48.5 nM). For PPQ, the median IC_50_ was 19.6 nM (range: 8.5–52.8 nM). Isolates had a median *ex vivo* IC_50_ value of 26.0 nM (range: 7.2–149.2 nM) to QN. Compared to the *ex vivo* assays performed in 2012–2015, we found a rising trend of IC_50_ for PND only (**Supplementary Table 3**). To detect whether the susceptibilities to different drugs were correlated, Spearman’s correlation analysis was performed between IC_50_s of pairs of the drugs. There is no correlation between the IC_50_ values of the seven antimalarial drugs of the parasite isolates in the study, except for a weak correlation between DHA and CQ (**Figure 1B**, r = 0.34, *P < *0.05).

**Table 1:** *Ex vivo* IC_50_ values (nM) of clinical P. vivax isolates to seven antimalarial drugs.

Drugs	Number	Median (IQR)	Range
Chloroquine	46	96.48 (77.15–131.30)	36.54–303.40
Artesunate	46	1.95 (0.99–3.30)	0.37–11.81
Dihydroartemisinin	46	1.63 (1.07–3.28)	0.34–10.15
Piperaquine	39	19.60 (13.30–24.20)	8.51–52.83
Pyronaridine	39	15.53 (11.22–21.99)	4.48–38.78
Mefloquine	39	16.38 (11.77–21.75)	5.75–48.46
Quinine	39	26.04 (15.96–47.91)	7.19–149.20

IQR–interquartile range.

### Polymorphisms in pvmdr1, pvmrp1, pvdhfr, and pvdhps Genes

The *pvmdr1*, *pvmrp1*, *pcdhfr*, and *pvdhps* genes were successfully sequenced in 46 P. *vivax* isolates. Compared with the Sal-I sequence, 13 non-synonymous substitutions (P8L, T409M, S513R, G520D, G698S, L845F, A861E, M908L, T958M, K997R, F1076L, K1393N, and S1450L) were identified in the *pvmdr1* gene (**Table 2**). Two substitutions M908L and T958M were fixed, while G698S was present in 97.8% of the parasite population. The two substitutions Y976F and F1076L, which might be associated with CQ resistance (Brega et al., 2005), had completely different prevalences: Y976F was not detected, whereas F1076L was highly prevalent (93.5%). A total of nine haplotypes *pvmdr1* were identified (**Table 3**). The most prevalent haplotype PTSG**S**LA**LM**K**L**KS (54.4%) carried four substitutions, followed by PTS**DS**L**ELM**K**L**K**L** (21.7%) with seven substitutions.

**Table 2:** The prevalence of *pvmdr1*, *pvmrp1*, *pvdhfr* and *pvdhps* genes substitutions in 46 parasite isolates and association of the mutations with altered *ex vivo* drug susceptibilities.

Gene/Polymorphisms	Number (%)	Association with altered *ex vivo* drug susceptibilities
*pvmdr1*		
P8L	1 (2.17)	Increase to CQ, AS and DHA
T409M	2 (4.35)	
S513R	3 (6.52)	
G520D	10 (21.71)	Decrease to PND
G698S	45 (97.83)	Decrease to CQ, AS and DHA
L845F	1 (2.17)	Increase to DHA and PPQ; Decrease to PND and MFQ
A861E	11 (23.91)	
M908L	46 (100.00)	
T958M	46 (100.00)	
K997R	2 (4.35)	
F1076L	43 (93.48)	
K1393N	3 (6.52)	
S1450L	12 (26.09)	
*pvmrp1*		
Y1393D	46 (100.00)	
G1419A	1 (2.17)	Increase to CQ; Decrease to AS, DHA, PPQ and MFQ
V1478I	45 (97.83)	Decrease to CQ, AS and DHA
*pvdhfr*		
F57I/L	5 (10.87)	
S58R	7 (15.22)	
T61M	5 (10.87)	
H99S	26 (56.52)	
S117N/T	8 (17.39)	
*pvdhps*		
A383G	30 (65.21)	
A553G	4 (8.70)	
E571Q	2 (4.35)	

**Table 3:** Prevalence of *pvmdr1*, *pvmrp1*, *pvdhfr* and *pvdhps* genes haplotypes*.

Genes	Haplotype	Number (%)
*pvmdr1*(8/409/513/520/698/845/861/908/958/997/1076/1393/1450)	PTSG**S**LA**LM**K**L**KS	25 (54.35)
	PT**R**G**S**LA**LM**KFKS	3 (6.52)
	**L**TSGGLA**LM**K**L**KS	1 (2.17)
	PTSG**S**LA**LM**K**L**K**L**	2 (4.35)
	PTSG**S**LA**LM**K**LN**S	1 (2.17)
	PTSG**S**L**ELM**K**L**KS	1 (2.17)
	PTSG**SF**A**LM**K**L**KS	1 (2.17)
	P**M**SG**S**LA**LM**K**LN**S	2 (4.35
	PTS**DS**L**ELM**K**L**K**L**	10 (21.74)
*pvmrp1* (1393/1419/1478)	**D**GV	1 (2.17)
	**D**G**I**	44 (95.65)
	**DAI**	1 (2.17)
*pvdhfr* (57/58/61/99/117)	FSTHS	13 (28.26)
	FST**S**S	25 (54.35)
	FSTH**T**	1 (2.17)
	F**R**TH**N**	1 (2.17)
	F**R**T**ST**	1 (2.17)
	**IRM**H**T**	4 (8.70)
	**LRM**H**T**	1 (2.17)
*pvdhps* (383/553/571)	AAE	16 (34.78)
	**G**AE	24 (52.17)
	**GG**E	4 (8.7)
	**G**A**Q**	2 (4.35)

*Mutations are highlighted in bold.

For part of the *pvmrp1* gene (497 bp), compared with the Sal-I sequence, three non-synonymous substitutions (Y1393D, G1419A, and V1478I) were identified (**Table 2**). The Y1393D substitution was fixed, and G1419A was highly prevalent (97.8%). The most prevalent haplotype was **D**G**I** (95.7%) (**Table 3**).

Mutations in *pvdhfr* at codons 57, 58, 61, 99, and 117 were detected in 10.9, 15.2, 10.9, 56.5, and 17.4% of the isolates, respectively. No mutations were found at positions 13 or 173 (**Table 2**). The most prevalent haplotype FST**S**S (54.4%) carried one mutation, which was followed by the wild type (28.3%) (**Table 3**). Three different tandem repeat variations were found in the *pvdhfr* gene. Type 1 was identical to the Sal-I reference sequence, type 2 had the H99**S** mutation, and Type 3 carried a deletion of six amino acids at positions 98–103 (THGGDN) (**Supplementary Figure S2**). Type II was the most common at 56.5% (26/46), followed by Type III at 28.3% (13/46). Of note, Type 3 carried only the S117**N** mutation. Compared with *pvdhfr*, *pvdhps* showed a relatively lower prevalence of mutation genotypes. A553G and E571Q were present at low frequencies (**Table 2**), A383G was detected in more than half of the isolates. The most prevalent haplotype **G**AE (52.17%) carried one mutation, which was followed by the wild type (34.8%) (**Table 3**).

### Correlation Between *pvmdr1* and *pvmrp1* Gene Polymorphisms and *Ex Vivo* Drug Susceptibilities

**Figure 2:**
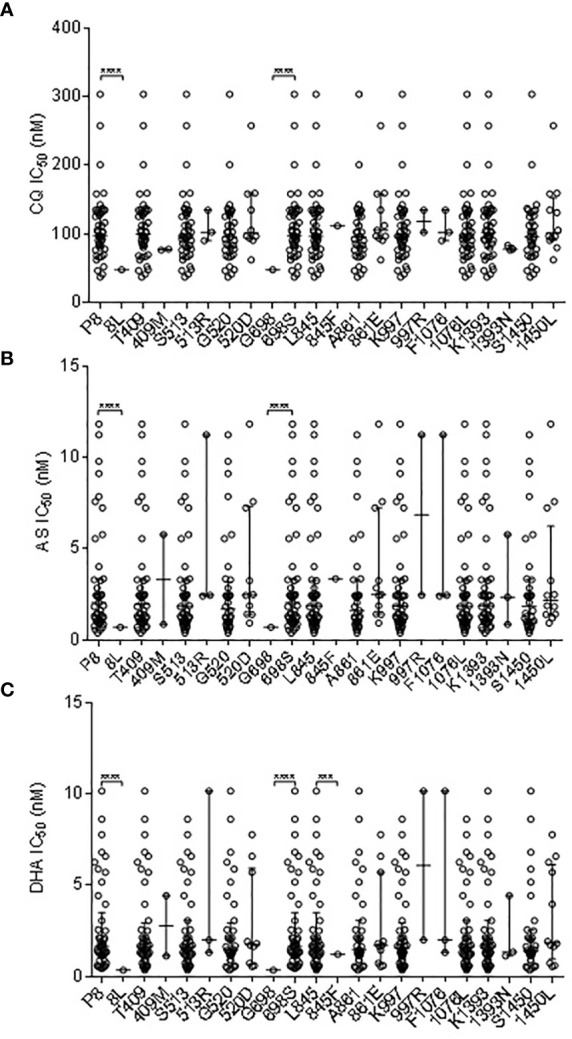
Correlation Association of SNPs in *pvmdr1* with *ex vivo* susceptibilities to CQ **(A)**, AS **(B)**, and DHA **(C)**. *** and **** represent indicate significant differences between the two alleles at *P* < 0.01 and <0.0001, respectively.

Of the 13 non-synonymous *pvmdr1* SNPs (P8L, T409M, S513R, G520D, G698S, L845F, A861E, M908L, T958M, K997R, F1076L, K1393N, and S1450L), P8L was significantly associated with the decreased IC_50_ to CQ, and G698S had a significant association with increased IC_50_ to CQ (P *< *0.05; **Figure 2**). Based on the cutoff IC_50_ value of 220 nM used by others (Suwanarusk et al., 2007), two *P*. *vivax* isolates were categorized as CQR. P8L had a significant association with the decreased IC_50_ to AS, and G698S had a significant association with the increased IC_50_ to AS (*P < *0.01; **Figure 2**). P8L and L845F had a significant association with the decreased IC_50_ to DHA, and G698S had a significant association with the increased IC_50_ to DHA (*P < *0.01; **Figure 2**). L845F had a significant association with the decreased IC_50_ to PPQ (*P < *0.01; **Supplementary Figure S2**). G520D and L845F had a significant association with the increased IC_50_ to PND (*P < *0.05; **Supplementary Figure S2**). L845F also had a significant association with the increased IC_50_ to MFQ (*P < *0.01; **Supplementary Figure S2**).

**Figure 3:**
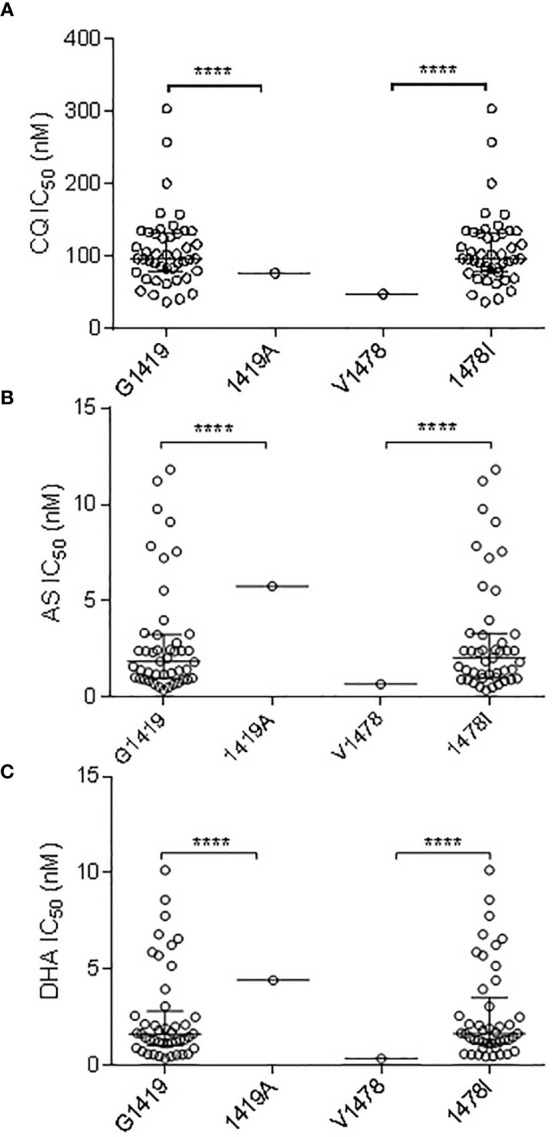
Correlation Association of SNPs in *pvmrp1* with *ex vivo* susceptibilities to CQ **(A)**, AS **(B)**, and DHA **(C)**. **** represents indicate significant differences between the two alleles at *P* < 0.0001.

Of the three non-synonymous *pvmrp1* mutations (Y1393D, G1419A, and V1478I), G1419A and V1478I had a significant association with the IC_50_ to CQ (*P* *< *0.01; **Figure 3**). In addition, the G1419A and V1478I substitutions were associated with the decreased susceptibilities to AS and DHA (*P < *0.01, **Figure 3**). G1419A was also associated with the decreased susceptibilities to PPQ, MFQ, and QN (*P < *0.01; **Figure 3**).

## Discussion

Drug resistance is a crucial issue for malaria control and prevention. In many malaria-endemic areas, CQ is still the drug of choice for the treatment of vivax malaria. CQR *P. vivax* was first reported from Papua New Guinea in 1989 (Rieckmann et al., 1989), followed by many *P*. *vivax* endemic areas, including the GMS (Cooper, 1994; Marlar et al., 1995; Singh, 2000; Bright et al., 2014; Shalini et al., 2014; Thanh et al., 2015; Baird, 2017). In the GMS, the high prevalence of mixed *P. falciparum* and *P. vivax* infections suggests that the extensive use of ACTs might have an exerted collateral selection pressure on the sympatric *P. vivax* populations. Here, we examined the *ex vivo* sensitivity of the *P. vivax* isolates collected from the China–Myanmar border area to seven commonly used antimalarial drugs. The overall result is that for most drugs, the IC_50_ values had a wide range, indicating the existence of parasites with reduced susceptibilities to commonly used antimalarials. For the correlation analysis of the antimalarial drugs, we did not find a significant correlation except for a weak correlation between DHA and CQ. This was consistent with our previous study, which found a weak correlation between CQ and AS (Li et al., 2020). Although this may not mean a similar resistance mechanism, it is noteworthy that these drugs are the most frequently used frontline treatment for *P. vivax* and *P. falciparum*, respectively, in the study area. Comparison with other *ex vivo* testing conducted in other endemic areas such as central China (Lu et al., 2011a), Thailand (Tjitra et al., 2008), South Korea (Chotivanich et al., 2009), Colombia (Fernández et al., 2014), and Indonesia (Pava et al., 2016), the CQ IC_50_ value for the China–Myanmar border isolates was lower than Thailand and Indonesia but higher than the IC_50_ value for parasites from the other regions. Due to the lack of a defined cutoff value for *P. vivax* CQ resistance, some researchers used 100 nM, while others used 220 nM as the cutoff IC_50_ value for CQ resistance (Suwanarusk et al., 2007; Barnadas et al., 2008). Using 220 nM as the cutoff value, we found that 4.3% (2/46) of the *P*. *vivax* isolates from the China-Myanmar border might be considered CQR, although this proportion was lower than our previous report (Li et al., 2020). Nevertheless, this result is consistent with our *in vivo* study on CQ efficacy in *P. vivax* patients in the same study area, where 2.6–5.2% of patients developed recurrent parasitemia within 28 days after CQ treatment, suggesting CQ resistance (Yuan et al., 2015; Xu et al., 2020). Regrettably, the clinical efficacy data of the parasite isolates used in this *ex vivo* study is lacking, so we cannot make further comparisons to obtain a more realistic resistance cutoff value for the parasites in this area. We also measured the *ex vivo* susceptibility of the parasite isolates to six other antimalarial drugs. For the artemisinin derivatives, the median IC_50_ values were relatively low, and did not correlate with drug susceptibilities to the quinoline drugs. This result is consistent with our previous report (Li et al., 2020), indicating that if *P. vivax* developed CQ resistance, ACT could be considered as an alternative for the treatment of CQR vivax malaria (Douglas et al., 2010; Baird, 2011). Compared with our previous study (Li et al., 2020), we found an upward trend of the IC_50_ values for PND between 2012 and 2016. While this may not indicate the emergence of PND resistance, this is coindental with the popular use of PND injections for malaria treatment in this region. For MFQ, the IC_50_ values were lower than those found in Thailand (Tjitra et al., 2008) and South Korea (Chotivanich et al., 2009), but close to Indonesia (Pava et al., 2016) and central China (Lu et al., 2011a), while the values were lower than those determined earlier at the same site (**Supplementary Table S3**) (Li et al., 2020). This appears consistent with that MFQ has not been used as the primary medicine for malaria in China and Myanmar.

There is no confirmed molecular marker for CQ resistance of *P. vivax* yet. *Pvmdr1* is often used to monitor CQ resistance assuming similarity to the *pfmdr1* gene, which mediates resistance to quinoline drugs in *P. falciparum*. In this study, sequencing of *pvmdr1* identified 13 non-synonymous substitutions, of which, G698S was associated with a reduced *ex vivo* CQ, AS, and DHA susceptibility. In addition, we found that the G520D and L845F were associated with a reduced susceptibility to PND, and that the L845F was also associated with a reduced susceptibility to MFQ. However, Y976F was not found in our study. This finding is consistent with other parasite populations from China and Myanmar (Nyunt et al., 2017; Wang et al., 2020), although it is often prevalent in different endemic areas, including Indonesia, Thailand, Cambodia, India, Papua New Guinea, and Ethiopia (Brega et al., 2005; Suwanarusk et al., 2007; Suwanarusk et al., 2008; Lu et al., 2011b; Golassa et al., 2015; Schousboe et al., 2015; Chaorattanakawee et al., 2017).

Population genomic analyses of *P. vivax* populations in Southeast Asia and Oceania found evidence that the *pvmrp1* gene is under a strong selection (Pearson et al., 2016; Benavente et al., 2017; Benavente et al., 2021). In South Asian populations, the *pvmrp1* gene exhibited high frequencies of two non-synonymous substitutions (D1393Y and G1419A). Similarly, this study detected fixation of the D1393Y mutation and G1419A approaching fixation (97.83%). Furthermore, we found that G1419A in our samples was associated with a reduced sensitivity to AS, DHA, PPQ, MFQ, and QN. It is noteworthy that V1478I was associated with a reduced *ex vivo* CQ, AS, and DHA susceptibility.

Our results indicate that *P. vivax* in the region was relatively resistant to the antifolate drug SP. Similar to *P. falciparum*, the resistance of *P. vivax* to antifolate drugs is related to mutations in the *pvdhfr* and *pvdhps* genes (Hawkins et al., 2007). Mutations at codons 50, 58, 117, and 173 of the *pvdhfr* gene confer resistance to pyrimethamine (Leartsakulpanich et al., 2002). Double mutations (S58**R** and S117**N**) were associated with a high level of resistance in *P. vivax*, whereas quadruple mutations (F57**L**/**I**, S58**R**, T61**M**, and S117**T**) were associated with SP treatment failure (Tjitra et al., 2002; Hastings et al., 2005). In this study, point mutations at codons 57, 58, 61, 99, and 117 of the *pvdhfr* gene were detected in 10.9–56.5% of the isolates. Here, the prevalence of the *pvdhfr* double or quadruple mutations was much lower than that found along the Thailand border and other areas of Myanmar (Nyunt et al., 2017; Tantiamornkul et al., 2018), but close to that from southern China (Miao et al., 2010). The most prevalent haplotype of the *pvdhfr* was FST**S**S, consistent with our previous report (Zeng et al., 2021). Mutant tandem repeats are also suggested to be associated with *P. vivax* antifolate resistance, and the frequencies of Type II (H99S type) and Type III (deletion type) were 56.52 and 28.26%, respectively, as compared to Type II being the predominant mutation in isolates from central China (Huang et al., 2014). Nevertheless, the highest frequency of tandem repeat variants was for the wild type in southern Thailand and Xishuangbanna Prefecture, Yunnan Province, China (Huang et al., 2014; Noisang et al., 2019). In India and Cambodia, Type III (deletion type) was the most common (de Pécoulas et al., 2004; Prajapati et al., 2011). The prevalent haplotypes of the *pvdhps* were GAE and the wild type, which is consistent with our previous report (Zeng et al., 2021). Similar to *pvdhfr*, the *pvdhps* mutations, triple or quadruple mutation haplotypes were less common than those in southern Thailand and southern Myanmar (Tantiamornkul et al., 2018; Noisang et al., 2019), suggesting that the parasites from the China–Myanmar border regions were less resistant to SP than other sites of the GMS.

## Conclusions

The *ex vivo* assays showed 4.3% of the *P. vivax* parasites in the China–Myanmar border areas as potential CQR isolates, which supports the results from *in vivo* CQ efficacy studies. While we identified correlations of the *pvmdr1* G698S and *pvmrp1* V1478I mutations with increased CQ IC_50_ values, further studies are required to validate the finding. We also found an increasing trend of PND IC_50_ values from 2012 to 2016, suggesting the emergence of PND resistance in the *P. vivax* population. Continuous *in vivo* and *ex vivo* studies to monitor the susceptibilities of the parasites to antimalarial drugs are needed to ensure the effective management of P. vivax cases and elucidate the mechanisms of resistance.

## Data Availability Statement

The datasets presented in this study can be found in online repositories. The names of the repository/repositories and accession number(s) can be found below: GenBank (*pvdhps* gene accession numbers: MZ746778 - MZ746823, *pvdhfr* gene accession numbers: MZ746824 - MZ746869, *pvmdr1* gene accession numbers: MZ746870 - MZ746915 and *pvmrp1* gene accession numbers: MZ746916 - MZ746961).

## Ethics Statement

The studies involving human participants were reviewed and approved by Kunming Medical University. The patients/participants provided their written informed consent to participate in this study.

## Author Contributions

WZe participated in the data collection, analysis, interpretation, and manuscript preparation. HZ, WZh, QY, XinL, XiaL, MD, XW, CL, ZX, and XC participated in the laboratory procedures, data collection, and analysis. ZY and LC contributed to the conception and design of the study. All authors contributed to the article and approved the submitted version.

## Funding

This study was supported by the National Institute for Allergy and Infectious Diseases (U19 AI089672), The National Institute of Health (NIH), USA. ZY was funded by the National Science Foundation of China (31860604 and U1802286), Major science and technology projects of Yunnan Province (2018ZF0081), and International Science and Technology Cooperation-Yunnan International Science and Technology Cooperation Base (202003AE140004). XC and ZX were sponsored by the Yunnan Applied Basic Research Projects-Union Foundation (2018FE001-190 and 2019FE001-015, respectively). WZ was supported by the Education Department Fund of Yunnan Province (2019J1184).

## Conflict of Interest

The authors declare that the research was conducted in the absence of any commercial or financial relationships that could be construed as a potential conflict of interest.

## Publisher’s Note

All claims expressed in this article are solely those of the authors and do not necessarily represent those of their affiliated organizations, or those of the publisher, the editors and the reviewers. Any product that may be evaluated in this article, or claim that may be made by its manufacturer, is not guaranteed or endorsed by the publisher.
