# Supplementary material for: Molecular Surveillance and Ex Vivo Drug Susceptibilities of Plasmodium vivax Isolates From the China–Myanmar Border
Source: Front Cell Infect Microbiol. 2021 Nov 1;11:738075. doi: 10.3389/fcimb.2021.738075 (PMC8591282; doi:10.3389/fcimb.2021.738075)
Supplement: Supplementary file 1 [file Presentation_1.pptx]

## Slide 1
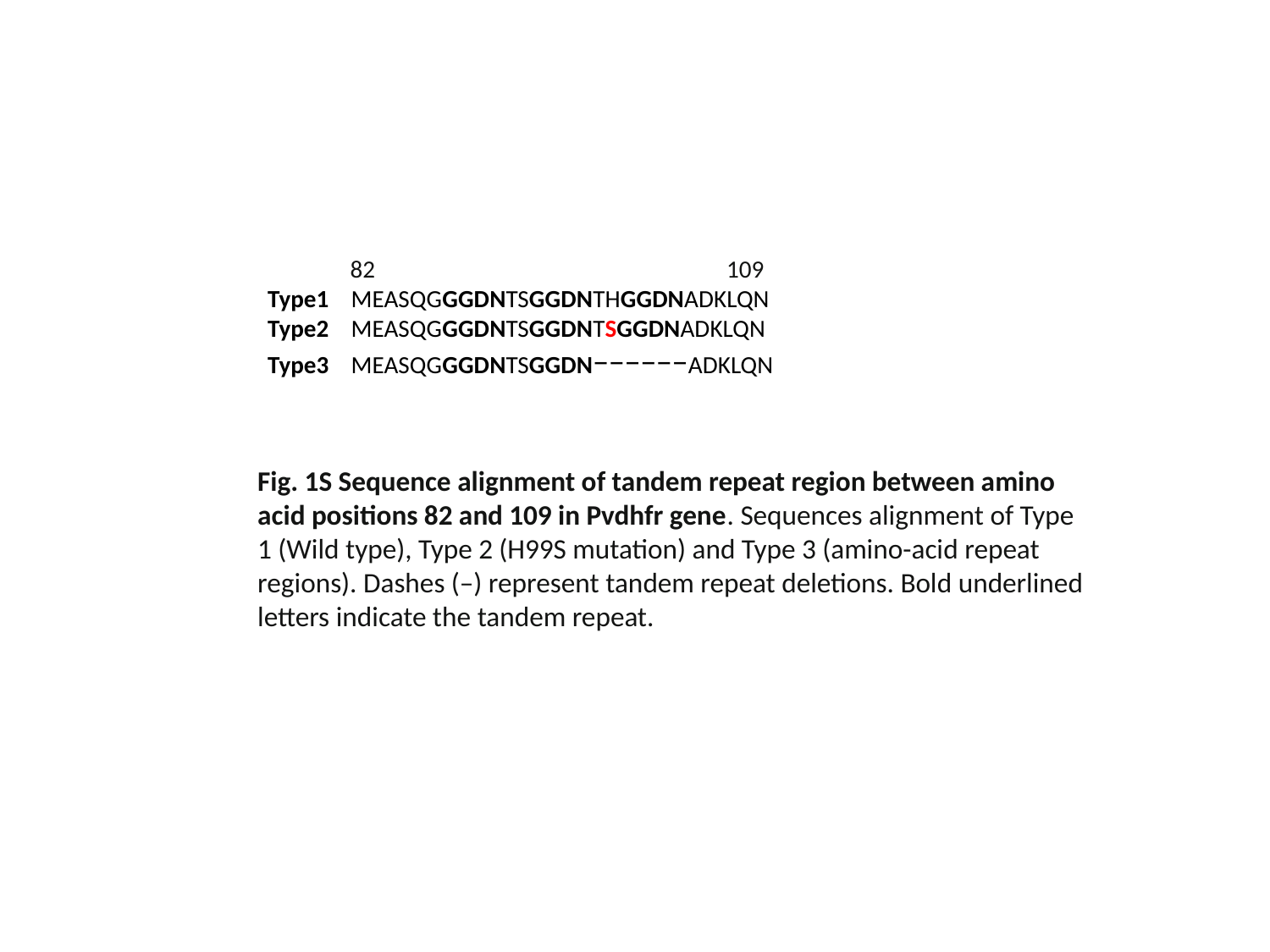

82 109
Type1 MEASQGGGDNTSGGDNTHGGDNADKLQN
Type2 MEASQGGGDNTSGGDNTSGGDNADKLQN
Type3 MEASQGGGDNTSGGDN−−−−−−ADKLQN
Fig. 1S Sequence alignment of tandem repeat region between amino acid positions 82 and 109 in Pvdhfr gene. Sequences alignment of Type 1 (Wild type), Type 2 (H99S mutation) and Type 3 (amino-acid repeat regions). Dashes (–) represent tandem repeat deletions. Bold underlined letters indicate the tandem repeat.

## Slide 2
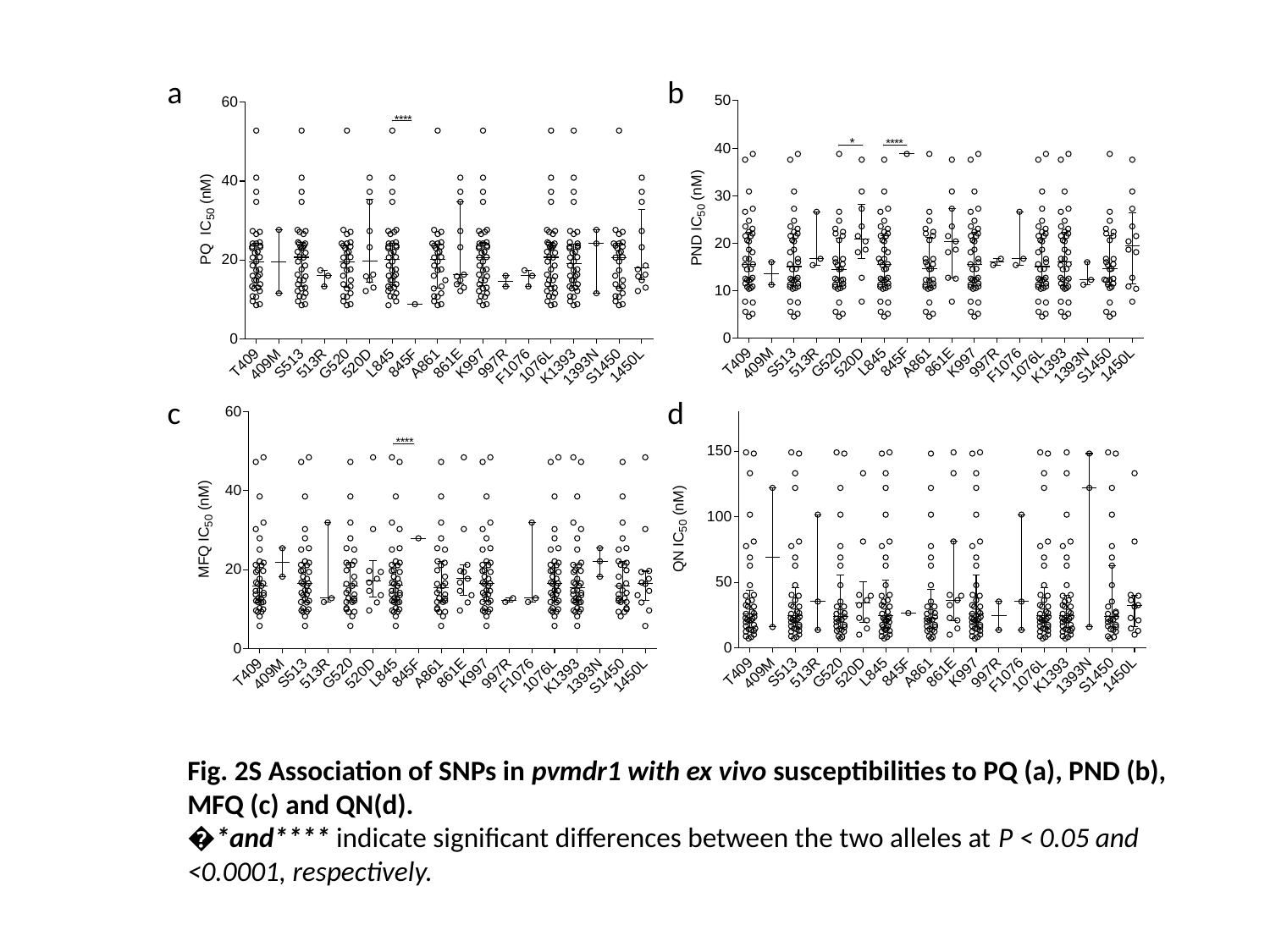

a
b
c
d
Fig. 2S Association of SNPs in pvmdr1 with ex vivo susceptibilities to PQ (a), PND (b), MFQ (c) and QN(d).
�*and**** indicate significant differences between the two alleles at P < 0.05 and <0.0001, respectively.

## Slide 3
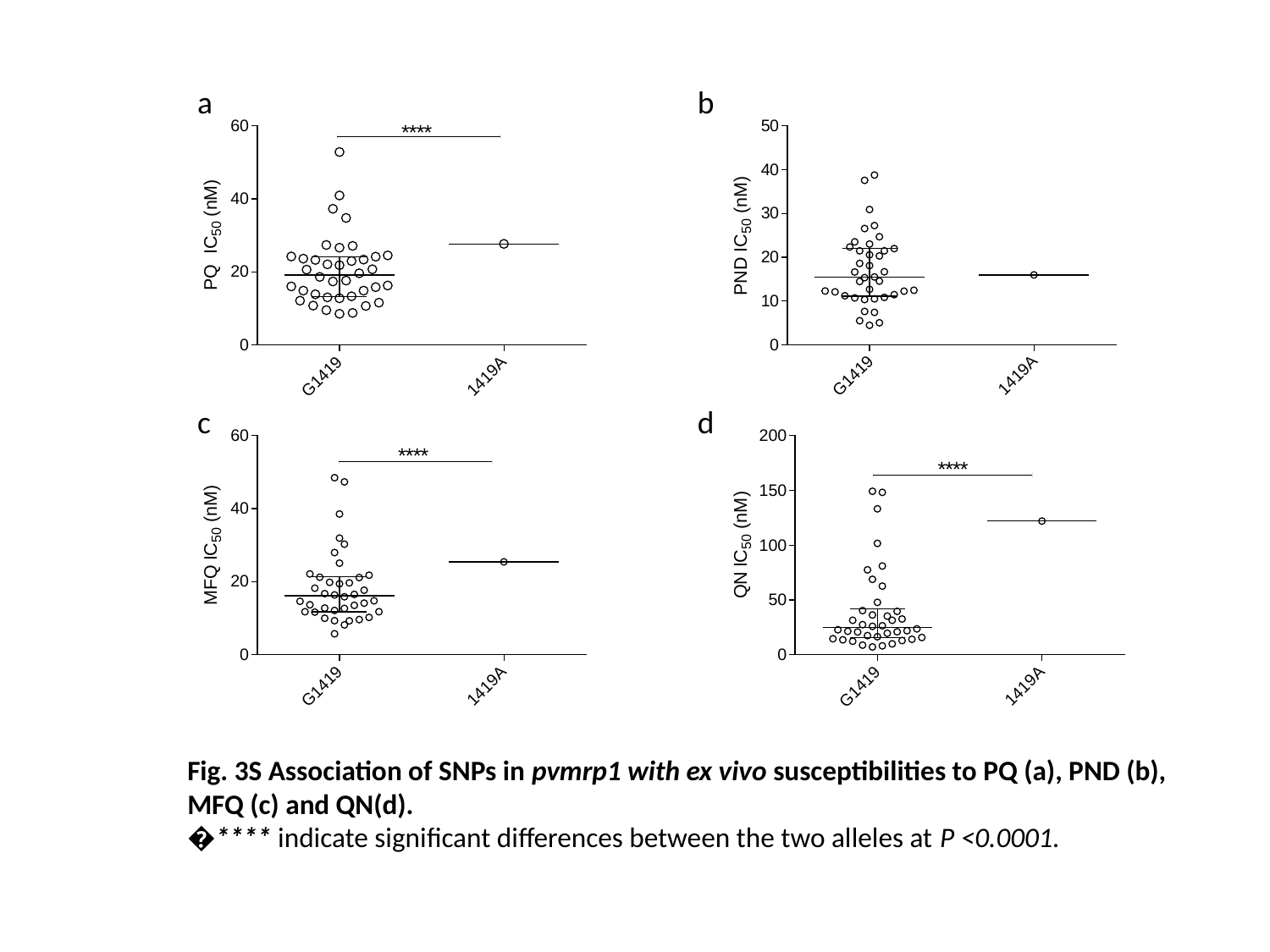

a
b
c
d
Fig. 3S Association of SNPs in pvmrp1 with ex vivo susceptibilities to PQ (a), PND (b), MFQ (c) and QN(d).
�**** indicate significant differences between the two alleles at P <0.0001.
