# Supplementary material for: Molecular Surveillance and Ex Vivo Drug Susceptibilities of Plasmodium vivax Isolates From the China–Myanmar Border
Source: Front Cell Infect Microbiol. 2021 Nov 1;11:738075. doi: 10.3389/fcimb.2021.738075 (PMC8591282; doi:10.3389/fcimb.2021.738075)
Supplement: Supplementary file 2 [file DataSheet_1.docx]

Supplement Table S1. Characteristics of *P. vivax* patients

| Patient feature | Value |
| --- | --- |
| Total no. of patients (% male) | 46 (67.4) |
| Median age (year) (range) | 21 (7–51) |
| Mean temperature (°C) (range) | 37.1 (36–39) |
| Malaria history within the past 12 months |  |
| Yes | 0 (0%) |
| No | 46 (100%) |
| Parasite density (/μl) (mean ± STD [range]) | 4654 ± 393.7 (760–12000) |

Supplement Table S2. PCR primer sequences for the amplification of *P. vivax* *pvmrp1*, *pvdhfr* and *pvdhps* sequences.

| Target gene |  | Primer sequence 5'–3' | Product size（bp） | Tm (^o^C) | Reference |
| --- | --- | --- | --- | --- | --- |
| *pvmrp1* | Fragment 1 | F1 ACGACATGATCCAAACGACA | 523 | 58 | (Chehuan et al., 2013) |
|  |  | R1 CTTATATACGCCGTCCTGCAC |  |  |  |
|  | Fragment 2 | F2 GGATAGTCATGCCCCAGGATTG | 497 | 58 |  |
|  |  | R2 CATCAACTTCCCGGCGTAGC |  |  |  |
| *pvdhfr* | Nested 1 | F1 ACCCTTCCATAGGGAGTCCACTT | 961 | 53 | (Mint Lekweiry et al., 2012) |
|  |  | R1 CGCATTGCAGTTCTCCGAA |  |  |  |
|  | Nested 2 | F2 CCCCACCACATAACGAAGTAG | 632 | 65 |  |
|  |  | R2 GCCGTTGATCCTCGTGAAG |  |  |  |
| *pvdhps* | Nested 1 | F1 GGAAGCCATTCGCTCAACTTATAA | 970 | 53 | (Mint Lekweiry et al., 2012) |
|  |  | R1 CGTCAGTTTACCCTCCCCGTT |  |  |  |
|  | Nested 2 | F2 GATGGCGGTTTATTTGTCGAT | 767 | 65 |  |
|  |  | R2 GCCTCCCCGCTCATCAGTCT |  |  |  |

Supplement table 3. *Ex vivo* IC_50_ values (nM) of clinical P. vivax isolates from 2012-2016

| Drugs | 2012 - 2013 | | | 2015 | | | 2016 | | | *P* value |
| --- | --- | --- | --- | --- | --- | --- | --- | --- | --- | --- |
|  | Number | Median (IQR) | Range | Number | Median (IQR) | Range | Number | Median (IQR) | Range |  |
| CQ | 40 | 86.9 (52.1 - 211.0) | 2.1 - 710.8 | 24 | 81.4 (21.0 - 173.4) | 9.9 - 319.4 | 46 | 96.5 (77.2 - 131.3) | 36.5 -303.4 | 0.6305^a^ |
| AS | 35 | 2.9 (1.7 - 4.8) | 0.1 - 6.7 | 24 | 2.1 (0.7 - 5.0) | 0.4 - 17.6 | 46 | 1.95 (1.0 - 3.3) | 0.4 -11.8 | 0.6174^a^ |
| DHA | 34 | 2.1 (1.2 - 3.6) | 0.2 - 11.5 | 24 | 1.8 (0.8 - 6.1) | 0.4 - 43.6 | 46 | 1.6 (1.1 - 3.3) | 0.3 - 10.2 | 0.2253^a^ |
| PQ | Not done | | | 24 | 22.3 (11.2 - 109.5) | 2.9 - 429.8 | 39 | 19.6 (13.3 -24.2) | 8.5 - 52.8 | 0.3622^b^ |
| PND | 40 | 2.9 (1.9 - 4.6) | 0.6 - 14.5 | 24 | 11.8 (6.4 - 64.3) | 2.3 - 97.3 | 39 | 15.5 (11.2 - 22.0) | 4.5 - 38.8 | < 0.0001^a^ |
| MFQ | 34 | 32.9 (25.7 - 45.8) | 5.7 - 335.6 | 24 | 39.0 (21.9 - 56.3) | 6.5 - 160.8 | 39 | 16.4 (11.8 - 21.8) | 5.8 - 48.5 | < 0.0001^a^ |
| QN | Not done | | | 24 | 41.4 (18.0 - 95.5) | 6.1 - 203.7 | 39 | 26.0 (16.0 - 47.9) | 7.2 - 149.2 | 0.1359^b^ |

^a^ used Kruskal-Wallis test, ^b^ used Mann Whitney test
